# Optimizing wheat production and reducing environmental impacts through scientist–farmer engagement: Lessons from the North China Plain

**DOI:** 10.1002/fes3.255

**Published:** 2020-11-04

**Authors:** Wei Jiang, Annah Zhu, Chong Wang, Fusuo Zhang, Xiaoqiang Jiao

**Affiliations:** ^1^ National Academy of Agriculture Green Development Department of Plant Nutrition, College of Resources and Environmental Sciences China Agricultural University Beijing 100193 China; ^2^ Environmental Policy group Wageningen University Wageningen Netherlands

**Keywords:** multi‐objective, smallholder farmers, sustainable intensification, wheat production

## Abstract

Producing high economic benefits and high grain yields with limited environmental impacts is crucial for feeding the world's growing population. Yet it remains challenging to improve the performance of one objective without creating unintended consequences for other objectives. This is especially difficult for smallholders navigating a diverse array of environmental and personal demands. This study demonstrates how combining participatory research through the Science and Technology Backyards (STB) approach with Pareto‐based ranking modeling can increase smallholder production while also reducing environmental impact. Through an intensive farmer survey in a 1 × 1 km grid in Quzhou County, we demonstrate that farmers engaged in STBs performed better according to multiple objectives (i.e., optimizing overall grain yield, benefit‐cost ratio, and GHG emissions, without compromising any one of these objectives) than farmer's not engaged in STBs. Moreover, we used a Pareto optimization approach (OPT) to determine the optimal smallholder scenario. We found that under OPT, grain yield could reach 9.5 t/ha, with a benefit‐cost ratio of 2.1, a 100% N recovery efficiency, and 7,395 kg CO_2_eq ha^−1^ GHG emissions. With OPT as a final goal, our research team worked with STB farmers to improve economic and environmental outcomes without compromising yield. Our findings demonstrate that no significant difference was obtained between farmers engaged in STBs and these under OPT. Compared with non‐STB farmers, STB farmers’ grain yield improved by 18%, benefit‐cost ratio improved by 26% due to improved N recovery efficiency, and GHG emissions were reduced by 31%. These improvements demonstrate the power of scientist–farmer engagement for optimizing wheat production. Such engagement allows farmers to modify their agronomic practices to more closely match Pareto optimal conditions, thus improving environmental and economic benefits without compromising yield. Our results provide solid evidence of the potential for sustainable wheat production by combining modeling with participatory research.

## Graphical abstract

We found that smallholders engaged with scientists through STBs came closer to these Pareto optimal conditions on average than smallholders. These results show that multi‐objective optimization in wheat production can be achieved by modifying agronomic practices through engagement between scientists and farmers. These improvements demonstrate the power of scientist–farmer engagement for optimizing wheat production. Such engagement allows farmers to modify their agronomic practices to more closely match Pareto optimal conditions, thus improving environmental and economic benefits without compromising yield. Our results provide solid evidence of the potential for sustainable wheat production by combining modeling with participatory research.

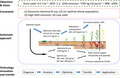

## 1 INTRODUCTION

In the past several decades, China has produced enough food to feed 22% of the global population with less than 9% of the global arable land (Fan et al., 2012). However, this so‐called miracle has also been accompanied by an enormous amount of resource and environmental costs (Jiao et al., 2016). As much as 35% of the chemical nitrogen (N) produced worldwide has been consumed for grain production in recent years (IFA, 2019). In the future, more than double the current grain production will be needed for the growing population (Godfray et al., 2010; Tilman et al., 2011). Feeding a large population in a sustainable manner rather than using a resource‐intensive approach is a great challenge faced by China's agriculture (Zhang et al., 2014, 2015). The challenges are more daunting in smallholder farmer‐dominated systems. On the North China Plain, for example, smallholders have produced 25% of the food with 26% of the arable land, a much less favorable ratio than for the country as a whole (NBSC, 2019). Having overcome the problem of hunger, China is now considering efficiency and the environment. Specifically, what is the best way to achieve multiple objectives (high economic returns, high yields, high N use efficiency, and low environmental impacts) rather than just maintaining high yield? Improving N use efficiency and other smallholder agronomic practices are the best ways to optimize production both economically and environmentally.

Many attempts have been made to achieve more with less resources in crop production (Chen et al., 2014; Zhang et al., 2016). Yet, this almost inevitably entails trade‐offs or is not feasible under real‐world conditions. For instance, Cui et al. (2008) found that employing an in‐season root‐zone N management strategy based on the soil N_min_ test can improve N use efficiency and farmer incomes and reduce GHG emissions. Similarly, Chen et al. (2014) found that an integrated crop‐soil management strategy can improve grain yield by 30% without increasing N use (Chen et al., 2014). While this has provided valuable research for sustainable crop production, real‐time in situ field monitoring is required in both cases, which has hindered widespread application of these techniques by smallholder farmers. In addition, these outcomes are not independent of each other. They interact in both positive and negative ways, creating the potential for synergies and trade‐offs (Groot et al., 2012).

In recognition of these trade‐offs, attempts have been made to develop models and decision support tools to help smallholder farmers select solutions for multi‐objective optimization in crop production from a top‐down perspective (Todman et al., 2019; Khoshnevisan et al., 2020). Pareto‐based multi‐objective optimization approaches have attracted great interest for solving such complicated problems. This approach can identify optimal solutions where any one indicator cannot be improved further without compromising the performance of the other indicators (Groot et al., 2012). Pareto optimization can provide a set of mathematically equivalent solutions from a large number of options. For instance, with a multicriteria evolutionary‐based algorithm, Khoshnevisan et al. (2020) recently developed a regional‐scale decision support system to optimize N use such that crop yield was maximized and negative environmental impacts were minimized. Indeed, exactly what smallholder farmers are capable of achieving in their own fields and the potential adaptive agronomic practices needed for such achievements are not fully understood.

Crop production is inherently a very complex process that includes land preparation, chemical fertilizer use, and pesticides (George et al., 2014). In the real world, an improvement in one objective is typically associated with negative effects on other objectives. For instance, high‐yield crop production is often associated with high chemical fertilizer use, while high chemical fertilizer use is usually accompanied by low economic benefits and low environmental quality (Jiao et al., 2016; Ju et al., 2016). A simple and effective approach that considers multiple objectives from a bottom‐up perspective is urgently needed. Stuart et al. (2018), for example, demonstrated that adoption of improved agronomic practices can increase profit and reduce excessive inputs, thus improving nutrient use efficiency and reducing environmental impacts. Kanter et al. (2016) further showed that that although scientifically sound technology application approaches were effective in helping smallholder farmers achieve multiple objectives in crop production, due to the lack of participation of the appropriate stakeholders in the research design and the generic solutions provided, the uptake in practice was quite low. In order to address these shortcomings, the objectives of the present study are as follows: (a) to explore Pareto optimal solutions for wheat production (optimizing wheat yield, N use efficiency, GHG emissions, and benefit‐cost ratio, without compromising any one of these objectives) and (b) to identify how optimal solutions can be achieved in practice through participatory approaches.

One method of participatory engagement of particular concern for our study is the Science and Technology Backyard (STB) model. The STB model was established in 2010 in Quhzou County of the North China Plain and has since gone on to cover different ecological zones across China (Zhang et al., 2016). The goal of STBs is to link the scientific community with the farming community through participatory research with the ultimate aim of empowering smallholders to achieve higher crop yields with less environmental impacts (Jiao et al., 2019). Through this approach, scientists and farmers conduct field trails and demonstrations to illustrate best agronomic practices and new technologies. With scientist support, farmers then conduct field trials in order to determine how to practically implement these best practices and technologies in their own fields. Our study combines this participatory approach with Pareto optimality modeling in order guide scientist farmer engagement with Pareto optimal conditions as an end goal. Through a comprehensive survey and follow‐up participatory research, we compare the production data of smallholder farmers engaged in STBs with smallholder farmers that are not, as discussed further below. This will enable us to, as discussed in the results and conclusion, determine the potential of the STB approach to optimize environmental and economic objectives by combining participatory research with Pareto‐based ranking.

## 2 MATERIALS AND METHODS

### 2.1 System boundary and data collection

**FIGURE 1:**
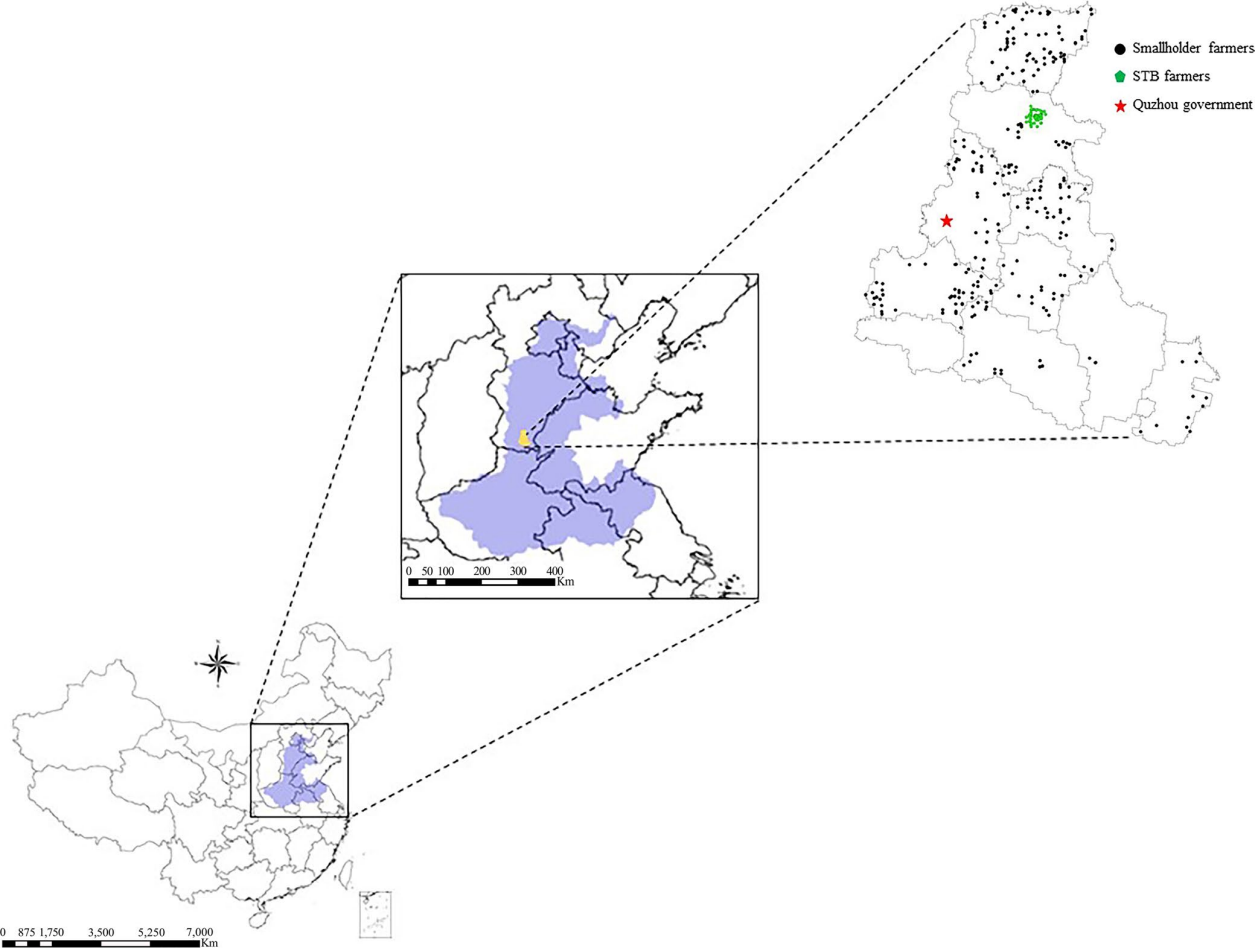
The distribution of farmers surveyed in the study. The blue zone on the left is the North China Plain. A total of 321 smallholder farmers were surveyed, including 73 STB farmers in Wangzhuang Village

An intensive farmer survey, covering 321 smallholders, was conducted in Quzhou County, Hebei Province (Figure 1). Quhzou County is a typical agricultural county in the North China Plain, demonstrating typical climate conditions and grain production patterns. The climate of this region is warm‐temperate, subhumid continental, and monsoonal with cold winters and hot summers. On average, the rainfall in the region is 500 mm per year, ranging from 400 to 700 mm. The average annual temperature is 13.1°C, ranging from −10 to 30°C. Wheat–maize rotation is the major cropping system in this zone. The growing period of wheat is normally from early October to June. During this time, a third of the annual rainfall occurs. As the bread‐basket of China, as much as 56% of the wheat grain in China is produced in this area. Smallholder farmers are the major force for wheat production in this area.

The survey was conducted over a 1 × 1 km grid of Quzhou County in March 2018. Of the 321 smallholder farmers participating in the survey, 73 farmers were part of an STB in Wangzhuang Village, a typical wheat–maize rotation village in Quzhou County (referred to as “STB farmers”) and 248 were not part of STBs (referred to as “FP farmers,” with FP standing for typical “farmer practices” in the region) (Figure 1). Farmer behaviors and agronomic practices, characteristics, and grain yield were asked and recorded through the survey, including the amount and timing of chemical fertilizer use (N, P_2_O_5_, and K_2_O), the number of wheat varieties used, farm size, sowing rate and date, and costs. The GHG emissions, benefit‐cost ratio, and N recovery efficiency were calculated according to the equations provided below. More information on the different farmer types (STB and FP farmers) and their agronomic practices are described in Table S1. Generally speaking, FP farmers employed typical farmer practices for the region. In contrast, STB farmers have been intensively monitored and trained since 2010, when the STB was established in Wangzhuang Village. According to the working approach of STB, scientists worked with farmers through jointly conducted field demonstrations and field trials to determine how to improve yield while reducing economic and environmental costs.

After conducting the farmer survey (including both FP and STB farmers), a Pareto‐based ranking approach was applied to the results (see Section 2.6 for a discussion of this approach). Through this methodology, farmers with Pareto optimal conditions (OPT) were identified. After identifying the Pareto optimal conditions, our research team continued to work with STB farmers in order to improve their agronomic practices to get closer to Pareto optimality. Through the typical STB approach but now also with Pareto optimality as an end goal, scientists worked with farmers to implement integrated agronomic practices that would allow farmers to come closer to Pareto optimal conditions. Working together, a desirable future of wheat production was envisaged and the barriers and constraints to achieve optimal wheat production were analyzed. With the end vision in sight, integrated agronomy practices were jointly developed. A set of field trials testing appropriate agronomic practices were conducted to develop these adaptive technologies in situ and field demonstrations were made to provide evidence of their effectiveness to other smallholders in the village. At the same time, intensive and long‐term training was performed, especially in key crop growth stages. Through this type of scientist–farmer engagement, recommended agronomic practices were implemented in a bottom‐up manner. By October 2018, after a year of participatory engagement with STB farmers following the initial survey, harvest data was collected for these STB farmers and used in our analysis (along with the survey data collected for FP farmers).

#### 2.1.1 N flow in wheat production

The N flow in wheat production was calculated using N inputs and outputs. The N input includes chemical N fertilizer (N_fert_) and N from deposition (N_dep_), irrigation (N_irr_), seeds (N_seed_), and biological fixation (N_bio_).

(1) $$N_{\text{input}}=N_{\text{fert}}+N_{\text{dep}}+N_{\text{irr}}+N_{\text{seed}}+N_{\text{bio}},$$

where N_fert_ was calculated as the amount of chemical fertilizer use multiplied by the concentration of N in the fertilizer. N_seed_ was calculated as the amount of seed used multiplied by the concentration of seed. N_dep_, N_irr_, and N_bio_ were the amount of N from deposition, irrigation water, and biological N fixation. The amount of chemical fertilizer and seed use was obtained from survey data. The concentration of chemical N fertilizer was collected from fertilizer bags labeled by the producers. The seed contribution was obtained from Yue et al. (2015), while N_dep_, N_irr_, and N_bio_ (calculated to be 21 kg N/ha, 13 kg N/ha, and 15 kg/ha, respectively) were obtained from Liu et al. (2014).

The N output includes N harvested in grain (N_up_), NH_3_ volatilization (N_NH3_), N leaching (N_leach_), N_2_O emissions (N_N2O_), and N accumulation in arable land (N_acc_). In Quzhou, all the straw was returned back to the soil.

(2) $$N_{\text{output}}=N_{\text{up}}+N_{\text{NH}3}+N_{\text{leach}}+N_{N2O}+N_{\text{acc}},$$

where N_up_ was calculated as the wheat yield multiplied by the grain N concentration. The wheat yield was obtained from survey data, and grain N concentration was obtained from Chen et al. (2014).

N_NH3_, N_leach_, and N_N2O_ were calculated as follows:

(3) $$N_{\text{NH}3}=0.17N_{\text{fert}}‐4.95,$$

(4) $$N_{\text{leach}}=13.59\times e^{\left(0.009\times \text{Nsurp}\right)},$$

(5) $$N_{N2O}=0.54\times e^{\left(0.0063\times \text{Nsurp}\right)},$$

where N_surp_ was calculated as the difference between N input (N_input_) and the N harvested as wheat grain (N_up_). The uncertainties of N flow in each type of farmers were listed in the Table S2.

### 2.2 Calculation of N recovery efficiency

The N recovery efficiency was calculated as the ratio between N input and N harvested as wheat grain (N_up_).

(6) $$N_{\text{effi}}=N_{\text{up}}/N_{\text{input}}\times 100\%,$$

### 2.3 Calculation of global warming potential (GWP) with life cycle analysis (LCA)

An LCA approach was employed to calculate the GHG emissions from wheat production. The functional unit is defined as the total GWP for wheat production per unit of arable land, expressed as kilograms of carbon dioxide equivalent per ha (CO_2_eq ha^−1^). The system boundaries were set from cradle to grave, including the burden of all material inputs and agricultural/industrial processes from wheat production.

The volatilization of compounds such as NH_3_ and NO*_x_* with their subsequent redeposition and leaching and runoff in wheat cultivation was estimated (Chen et al., 2014). Indirect N_2_O emissions can be estimated by following the IPCC methodology, where 1% and 0.75% of the volatilized N‐NH_3_ and leached N‐NO_3_ are lost as N_2_O−N, respectively. The GHG emissions from the total N_2_O emissions were calculated in units of CO_2_ equivalents (CO_2_ eq) over a 100‐year time period and were 298 times the intensity of CO_2_ on a mass basis. Using the above total N_2_O emissions per unit area, we calculated the GHG emissions, expressed as kg CO_2_ eq.

(7) $$\text{GHG}=\left({\text{GHG}}_{m}+{\text{GHG}}_{t}\right)\times N_{\text{fert}}+N_{N2O}\times 44/28\times 298+{\text{GHG}}_{\text{others}},$$

where GHG_m_ and GHG_t_ are GHG emissions from chemical N manufacturing and transportation per unit of chemical N fertilizer, expressed as kg CO_2_eq kg^−1^ N. GHG_others_ are GHG emissions from chemical P and K fertilizers, pesticides, herbicides, diesel consumption for irrigation, land preparation, and harvest in wheat production, including the inputs from the production, transportation, and application of these factors.

### 2.4 Benefit‐cost ratio analysis

The benefit‐cost ratio (BCR) was calculated as the ratio between costs (T_cost_) and profit (T_benefit_). The cost of wheat production was calculated by multiplying the unit price of the inputs by the amount of inputs. The input prices were calculated as an average of three years of prices in Quzhou County.

The costs of the system were calculated with the following equations:

(8) $$\begin{array}{cc} T_{\text{cost}}= & I_{\text{land}}\times P_{\text{land}}+I_{\text{electricity}}\times P_{\text{electricity}}\\ & \;+I_{\text{pesticides}}\times P_{\text{pesticides}}+I_{\text{seeds}}\times P_{\text{seeds}}\\ & \;+I_{N}\times P_{N}+I_{P}\times P_{P}+I_{k}\times P_{K}+I_{\text{diesel}}\times P_{\text{diesel}}, \end{array}$$

where I_i_ is the input for wheat production, and P_i_ is the unit price of the input.

(9) $$T_{\text{benefit}}=O_{\text{grain}}\times P_{\text{grain}},$$

where O_grain_ is the wheat yield and P_grain_ is the unit price of wheat.

$$\text{BCR}=T_{\text{benefit}}/T_{\text{cost}},$$

### 2.5 Optimization of objective functions

A generic multi‐objective linear programming model, called Pareto‐based ranking approach, was employed to explore the potential of wheat production while minimizing GHG emissions and maximizing wheat yield, benefit‐cost ratio, and N recovery efficiency in the 321 smallholder farms. This generic model, broadly covering the characteristics of the average farm, can be expressed in compact form as follows:

(10) $$\text{Max U}\left(x\right)={\left[U_{1}\left(x\right),U_{2}\left(x\right),U_{3}\left(x\right),‐U_{4}\left(x\right)\right]}^{T},$$

$$X={\left(x_{1},x_{2}\ldots x_{n}\right)}^{T},$$

Subject to *i* constraints:

(11) $$g_{i}\left(x\right)\leq h_{i},$$

where U_1_(*x*), U_2_(*x*), U_3_(*x*), U_4_(*x*) are the wheat yield, N recovery efficiency (Equation (6)), benefit‐cost ratio (Equation (10)), and GHG emission (Equation (7)), irrespectively. These objective functions that are simultaneously maximized or minimized, and (*X*_1_ … *X*_n_) are the decision variables that represent adjustable parameters to describe the adopted agronomic practices. The decision variables are the amount and timing of chemical N fertilizer use, the sowing rate, and so on. The constraints in Equation (12) can arise from the problem formulation, from limitations on the farm model results related to a specific configuration of the decision variables. It can be expressed as follows:

In maximizing the wheat yield and benefit‐cost ratio, the farm size cannot exceed the total farm area of 48,000 ha. Therefore, the cultivated area of wheat production should be less than or equal to the total arable land in whole county.

(12) $$\text{TPA}=\sum_{k=0}^{n}Ai\leq 48,000,$$

where TPA is the total planting area in ha. *A* is the planting area per smallholder.

The chemical N fertilizer use per unit of area cannot exceed the threefold of the amount of N harvested by wheat grain (Chen et al., 2014).

(13) $$N_{\text{fert}}\leq 3N_{\text{up}},$$

The topdressing N rate cannot exceed the total amount of chemical N fertilizer use in one growing season.

(14) $$N_{\text{top}}\leq N_{\text{fert}},$$

where N_top_ is the amount of N topdressing.

The first criterion for the performance of a case study is its Pareto rank, as proposed by Goldberg (1989). Individuals in the population are Pareto optimal when it is not possible to improve any one aspect of their performance, without compromising at least one of the other aspects. In such cases, there is no objective basis for discarding the individual. These individuals are referred to as nondominated and receive a rank of 1. In the present study, all farmers identified within the Pareto rank‐1 case (including both STB and FP farmers) are referred to as OPT farmers. The next step in Pareto‐ranking the entire population of solutions is to remove the individuals of rank 1 from the population and identify a new set of nondominated individuals that are assigned rank 2. This process is continued until all individuals in the population are assigned a Pareto rank. When information on the prior performance of the farming system is used, the ranking mechanism of Goldberg (1989) may be slightly adjusted to improve the selection of the part of the solution space where solutions are found that perform better than the original practices. In this case, a (superior) rank of 0 is assigned to solutions that perform better than the original configuration for all the objectives.

### 2.6 Data analysis

Data about wheat production and its corresponding agronomy practices was analyzed with one‐way ANOVA using SAS statistical software (SAS Inst.). Significant differences among means were determined by LSD at *p ≤ *.05. The data were presented by Sigma‐plot (version 12.0, Systat Software Inc.). N flow of wheat production under different farmer types was presented with e!Sankey (version 4.1, Hamburg, Germany).

## 3 RESULTS

### 3.1 N flow

**FIGURE 2:**
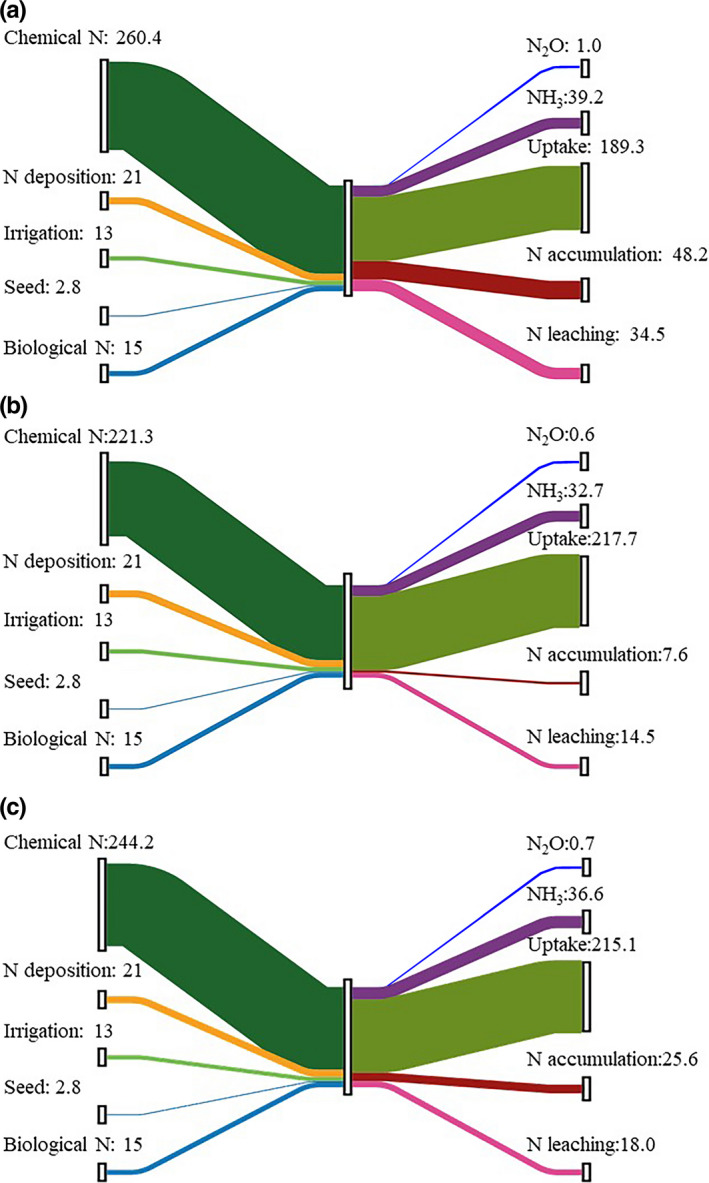
N flows from wheat production based on typical farmer practices (FP) (a), Pareto rank 1 farmers (OPT) (b) and STB farmers (STB) (c) in Quzhou County in 2017. A total of 321 smallholder farmers were surveyed in Quzhou County in 2017. Seventy‐three were STB farmers, while the remaining 248 were FP farmers. Of both STB and FP farmers, 33 were OPT farmers (7 STB farmers and 26 FP farmers)

For FP farmers, the total N input, including that from chemical fertilizer, irrigation, deposition, and biological fixation, was 312.2 kg N/ha, 83.4% of which was from chemical fertilizer. A total of 60.6% of N was used by the wheat grain. As much as 74.7 kg N/ha was lost to the environment (NH_3_ volatilization, N leaching, and denitrification), and 48.2 kg N/ha was accumulated in the arable land. Compared with that of FP, the total N input of the Pareto‐based farmers (OPT) was reduced to 273.1 kg/ha; the N uptake by wheat was increased by 15%; the N lost to the environment was reduced to 47.8 kg/ha; and only 7.6 kg N/ha was left in the arable land. For STB farmers, the N input was 296 kg/ha, and 55.3 kg/ha and 25.6 kg/ha were lost to the environment and accumulated in croplands, respectively (Figure 2).

### 3.2 Performance indicators for different types of farmers

**FIGURE 3:**
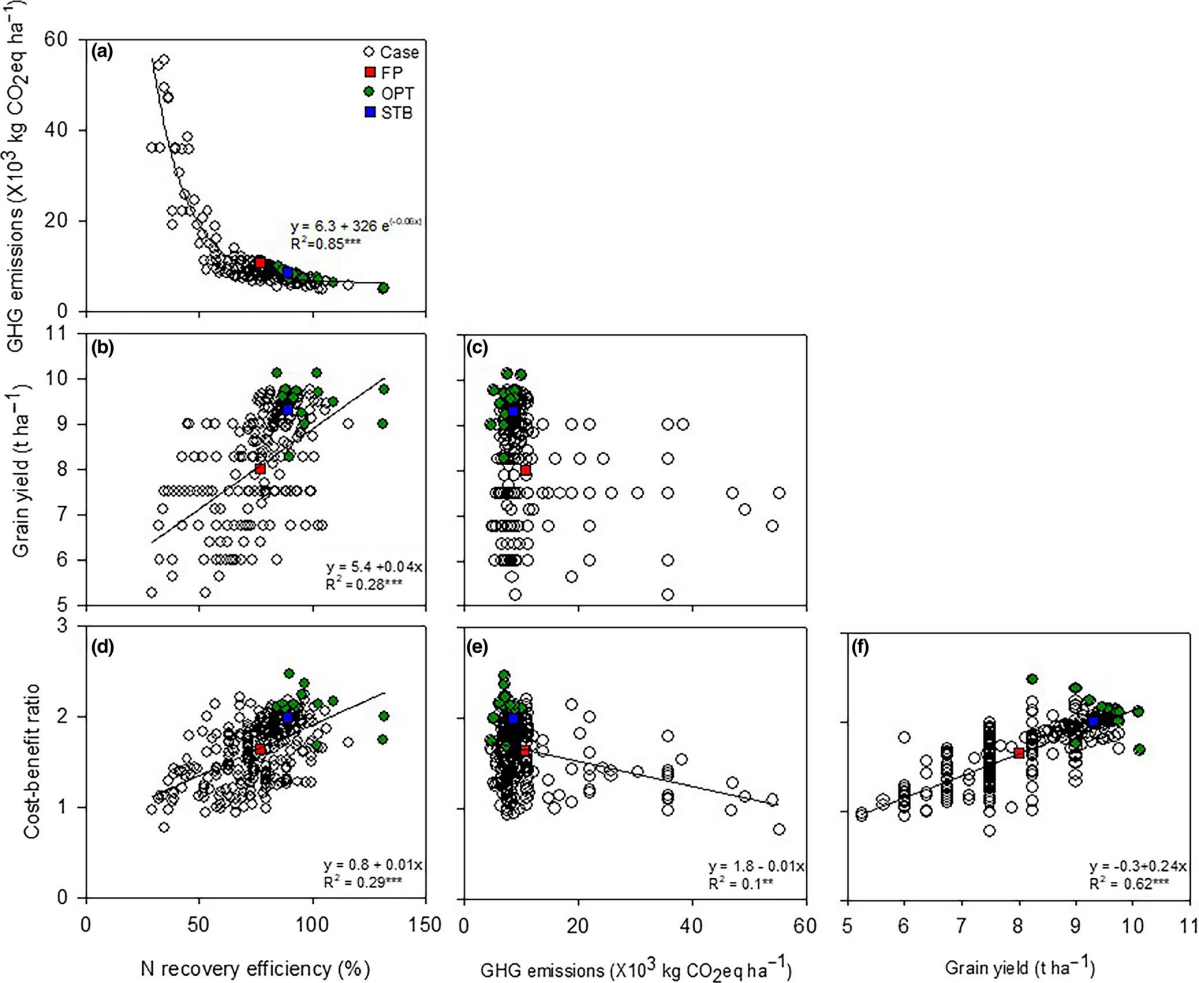
Relationships between wheat production performance indicators in Quzhou County in 2017. Each performance indicator is represented by Pareto frontiers after multi‐objective optimization for a full exploration of the trade‐off frontier. Each dot represents a performance configuration: green indicates Pareto rank 1 solutions (OPT), blue indicates the average value for STB farmers (STB), red indicates the average value for farmers employing typical farmer practices (FP), and white indicates all the cases (case). The formula presented in the graph indicates a significant relationship between objectives

An analysis of the relationship between the objectives of the smallholder farmers was performed (Figure 3). Synergy was observed between N recovery efficiency and grain yield as well as the benefit‐cost ratio (Figure 3b,d). The grain yield and benefit‐cost ratio also exhibited synergetic trends (Figure 3f). There was a large trade‐off between N recovery efficiency and GHG emissions (Figure 3a). A trade‐off between GHG emissions and the benefit‐cost ratio was also observed (Figure 3e). With the Pareto approach, 13 smallholder farmers that simultaneously achieved multiple objectives were selected among the 321 cases.

### 3.3 Characteristics of the 13 solutions near the extremes (minima or maxima) of the four objectives

**FIGURE 4:**
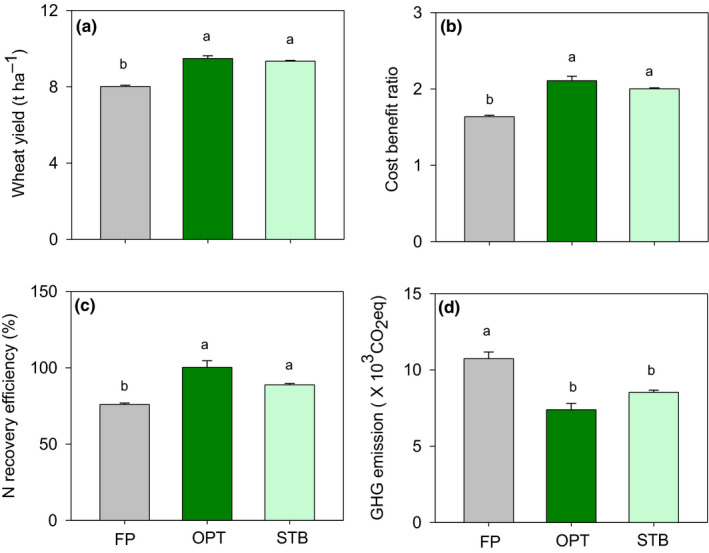
Wheat yield (a), cost‐benefit ratio (b), N recovery efficiency (c), and GHG emissions (d) in the three different study groups (farmer practices (FP), Pareto optimization (OPT), and STB farmers (STB)) in Quzhou County in 2017. Each value is the mean of cases (+*SE*). Different lower case letters denote significant difference (*p* ≤ .05) between categories

Compared with that under FP, the performance of wheat production in OPT improved greatly (Figure 4). The grain yield and cost‐benefit ratio in OPT were 9.5 t/ha and 2.1, respectively. The N recovery efficiency was improved by 32.1%, and GHG emissions were reduced by 31.1% compared with those under FP. A 9.2 t/ha wheat yield and 2.0 benefit‐cost ratio were obtained by STB farmers. N recovery efficiency in STB farmers was 100%, and GHG emissions were reduced by 15% compared to those under FP.

**FIGURE 5:**
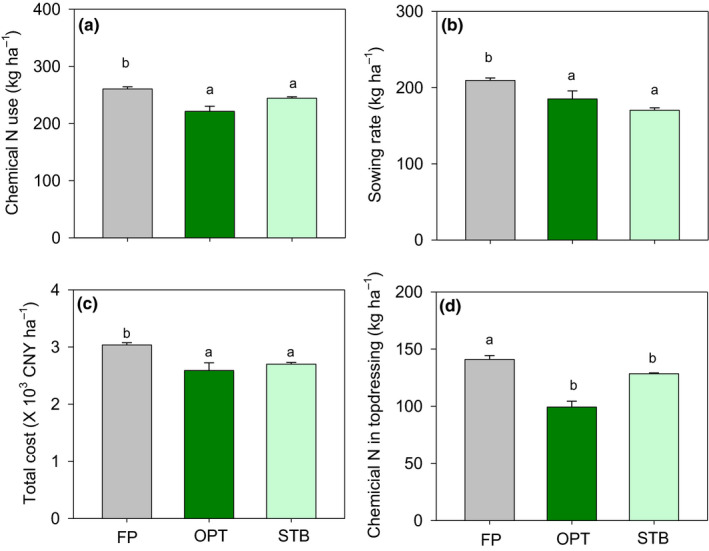
N application (a), sowing rate (b), total cost (c), and chemical N in topdressing (d) in the three different study groups (farmer practices (FP), Pareto optimization farmers (OPT) and STB farmers (STB)) in Quzhou County in 2017. Each value is the mean of cases (+*SE*). Different lower case letters denote significant difference (*p* ≤ .05) between categories

The key agronomic practices of OPT and STB farmers were improved (Figure 5). Chemical N fertilizer application was reduced from 260 kg/ha to 221 kg/ha and 250 kg/ha in OPT and STB farmers, respectively. Sowing rate was reduced by 15.7% and 14%, respectively, compared with that under FP. Total cost, including land preparation, labor, chemical fertilizer use and so on, was reduced by 7.4% and 8%, respectively, compared with that of FP.

The extreme of each objective and its corresponding agronomic practices were also evaluated (Table 1). The best results for individual objectives could only be reached at the expense of other objectives. For instance, when aiming to increase the maximum wheat yield from 8.0 t/ha under FP to 10.1 t/ha under OPT, chemical N use was project to be reduced from 260 kg/ha to 225 kg/ha, and the sowing rate should be reduced by 14.8%. At the same time, under these practices, N recovery efficiency increased by 34.4%, GHG emissions decreased by only 29.6%, and the benefit‐cost ratio was similar to that under FP. When the benefit‐cost ratio was maximized, the grain yield only increased by 15.4%, and the N recovery efficiency increased by 26.0% compared to those under FP; the total cost of wheat production was reduced by 17.5%, and chemical fertilizer input was reduced by 14.0%. When environmental concerns were given top priority, the GHG emissions were only 4,748 kg CO_2_eq ha^−1^, but large decreases in the grain yield and benefit‐cost ratio were observed. The grain yield was only improved by 1 t/ha compared with that under FP, and the benefit‐cost ratio was increased by only 6.7%; the chemical N input was as low as 159 kg/ha to achieve 9 t/ha wheat production.

**TABLE 1:** Characteristics of 7 solutions near the extremes (minima or maxima) of the four objectives

Items	Variable	Farmer practices	STB farmer		Highest wheat yield		Highest benefit‐cost ratio		Highest *N* recovery efficiency		Lowest GHG emissions	
			Value	Δ (%)	Value	Δ (%)	Value	Δ (%)	Value	Δ (%)	Value	Δ (%)
Objective	Wheat yield (t/ha)	8.0	9.2		10.1		9.2		9.8		9.0	
	Benefit‐cost ratio	1.6	2		1.7		2.2		2		1.8	
	*N* recovery efficiency (%)	75.9	87.0		102.1		95.7		132.0		131.2	
	GHG emissions (kg CO_2_eq ha^−1^)	10,737.3	8,586.1		7,558.8		7,283.6		5,165.9		4,748.4	
Agronomic practice	Cultivated area (mu)	9	8	−11 (±10.3)	12	33 (±15.4)	8	−11 (±10.3)	5	−44 (±6.4)	10	11 (±12.9)
	Wheat variety	3	3	0 (±0)	2	−33 (±0.5)	2	−33 (±0.2)	2	−33 (±0.4)	2	−33(±3)
	Sowing rate (kg/ha)	202.5	170.8	−15.7 (±9.3)	172.5	−14.8 (±8.4)	157.5	−22.2 (±16.4)	187.5	−7.4 (±1.8)	225	11.1 (±15.0)
	*N* application rate (kg/ha)	260.4	246.0	−5.5 (±19.2)	225	−13.6 (±17.6)	222.8	−14.5 (±17.4)	168.8	−35.2 (±13.2)	159	−38.9 (±12.4)
	Fertilizer cost (CNY ha^−1^)	3,034.1	2,633.2	−13.2 (±18.4)	3,900	28.5 (±27.3)	2,127.7	−29.9 (±14.9)	2,925	−3.6 (±20.4)	1,860	−38.7 (±13.0)
	Topdressing *N* rate (kg *N*/ha)	140.9	128.4	−8.9 (±4.0)	112.5	−20.1 (±10.8)	120.8	−16.6 (±7.2)	56.25	−60.1 (±9.8)	69	−51.0 (±10.9)

Values displayed are related to the objectives and decision variables for agronomic practices. Δ(%) indicates the relative change of horticulture practices for each extreme, compared with typical farmer practices (FP), with the standard deviation (*SD*) shown in parentheses. The underlined values indicate the magnitude of the improvement for the performance of each objective

## 4 DISCUSSION

### 4.1 Determining the optimal conditions for smallholder wheat production

Producing high yields and economic benefits while limiting environmental risks is one of the major challenges faced in grain production (Foley et al., 2011; Zhang et al., 2013). This challenge is even more daunting on the North China Plain due to the domination of wheat production by smallholders (Zhang et al., 2016). Smallholder farmers work at a scale at which it is difficult to realize and manage the trade‐offs between the potential benefits and negative impacts of their agronomic practices. Our study demonstrates the potential for optimizing smallholder wheat production while reducing environmental impacts. We do this by combining a Pareto‐based ranking approach with a substance flow analysis to determine the optimal solution to smallholder wheat farming when considering both economic and environmental factors.

Many attempts have been made to develop innovative solutions for sustainable crop production (Liu et al., 2016; Zhang et al., 2018). For instance, optimal N use and best N management practices have been developed by generating relationship curves between the N rate and specific indicators such as yield, economic income, and N uptake ( Wang, Ye, & Chen, 2014; Ying et al., 2017). Such an approach is helpful for developing an N management strategy. However, when trade‐offs or syntheses among different indicators exist (such as yield should be maximized while N loss should be minimized), it is difficult to make decisions using this approach. Therefore, meeting smallholder farmer demands from the perspective of both socioeconomic and environmental objectives, rather than one or the other, is vital for achieving the sustainable intensification of crop production.

Previous studies have showed that high‐yield crop production is often associated with high chemical N fertilizer use, resulting in low N recovery efficiency and a higher benefit‐cost ratio (Tilman et al., 2001; Zhang et al., 2015). However, in the present study, synergies were found between wheat yield and N recovery efficiency and the benefit‐cost ratio (Figure 3). This indicates that high yields and high N use efficiency in wheat production can be achieved in smallholder farmer plots, resulting in higher economic benefits. Similar results were obtained in previous studies on the North China Plain with improved wheat varieties and N management strategies in wheat production (Lu et al., 2016; Zhang et al., 2020).

In the present study, those farmers that demonstrated Pareto optimal socioeconomic and environmental factors (OPT) achieved an 18.4% improvement in grain yield and a 32.1% improvement in N recovery efficiency, figures comparable with high‐yield wheat production in the UK and showing even higher N recovery efficiency than US crop production (Zhang et al., 2015; Perryman et al., 2018). OPT farmers employed several key agronomic practices to increase their grain yield and N recovery efficiency. Compared with other farmers (FP), they reduced their sowing rate by 11.1% to avoid excessive population numbers in the early growth stage and to also avoid competition for the limited soil N in the root zone (Lu et al., 2015). Moreover, OPT farmers employed an optimal N use rate to spatially and temporally align the soil N supply in the root zone with wheat N demand (Shen et al., 2013). They reduced chemical N fertilizer use by 15.0% compared to FP farmers, and the proportion of topdressing by OPT farmers increased by 32.3%. Previous studies have shown that as much as 50% of the total chemical N fertilizer used could be reduced by optimized N use and split N application in crop production, thus improving N recovery efficiency to as high as 90% (Ju et al., 2009). OPT farmers averaged 213 kg N/ha, which is within the recommended range for chemical fertilizer use based on field trials on the North China Plain (Liu et al., 2016).

In the present study, high N recovery efficiency was associated with lower GHG emissions and higher economic benefits. Chemical fertilizer, especially N, is one of the greatest contributors to GHG emissions (Huang et al., 2017) and a large source of farmer input costs (Withers, Sylvester‐Bradley, Jones, Healey, & Talboys, 2014). On the North China Plain, approximately 50% of chemical N used by smallholder farmers is lost to the environment—10% as leaching, 30% as NH_3_, and 10% as runoff (Ju et al., 2016). In the present study, as much as 60% of the GHG emissions were generated from chemical N fertilizer use, which is in the range estimated by previous studies (Wang et al., 2017). Thus, we find that agronomic practices such as optimizing chemical N use and modifying chemical N types can improve N use efficiency and reduce GHG emissions. OPT farmers increased their benefit‐cost ratio by 25.6%, and GHG emissions by 31.1% due to the use of improved agronomic practices. Consequently, OPT farmers simultaneously achieved both high economic and environmental performance in wheat production.

### 4.2 Optimizing wheat production in practice through a participatory approach

It is one thing to identify the Pareto optimal solution for smallholder farming, it is quite another to implement this solution in the smallholders’ fields. This study demonstrates the efficacy of the STB model in helping smallholder farmers achieve Pareto optimal solutions on the ground. Specifically, it highlights the importance of scientist–farmer engagement to produce knowledge by combining Pareto‐based ranking modeling with a participatory approach. To the best of our knowledge, this is the first study to provide strong evidence for the success of such a combined approach. Compared with FP farmers, STB farmers improved the wheat yield, benefit‐cost ratio, and N recovery efficiency by 15.2%, 22.0%, and 14.5%, respectively, and reduced GHG emissions by 20.0% (Figure 4). These improvements are attributable to changes in agronomic practices, such as wheat management and soil nutrient management strategies (Table 1; Figure 5), that were determined by both scientists and farmers together, knowing the Pareto optimal solution and working backwards to achieve that solution.

Previous studies have shown that spatially optimal N application rates for different regions on the North China Plain were introduced through a multicriteria evolution‐based algorithm and provided a decision support system for policymakers (Khoshnevisan et al., 2020). This system has provided a valuable paradigm for sustainable wheat production. However, the applicability of this system was not tested in smallholder farmer field plots. Implementing multi‐objective optimization on the ground and translating it into smallholder farmer actions is a complicated process. It depends on a series of factors, such as human capital, risk preference, and geographic considerations (Feder and Umali, 1993; Mariano et al., 2012). Therefore, the routine application of these solutions in actual decision‐making has been limited (Groot et al., 2012; Kanter et al., 2016). Many factors can explain the low uptake rate of multi‐objective solutions. One important cause is the serious lack of participation in research design and research process by smallholder farmers (Kristjanson et al., 2009). Although scientists provide generic recommendations for sustainable crop production using formal logic, most solutions from scientists are not provided to a clear end‐user or stakeholder group (Sterk et al., 2011). Solutions have failed to bridge the knowledge/action gap because scientists often lack an understanding of the views of smallholder farmers, and smallholder farmers lack an interest in building partnerships with scientists.

**FIGURE 6:**
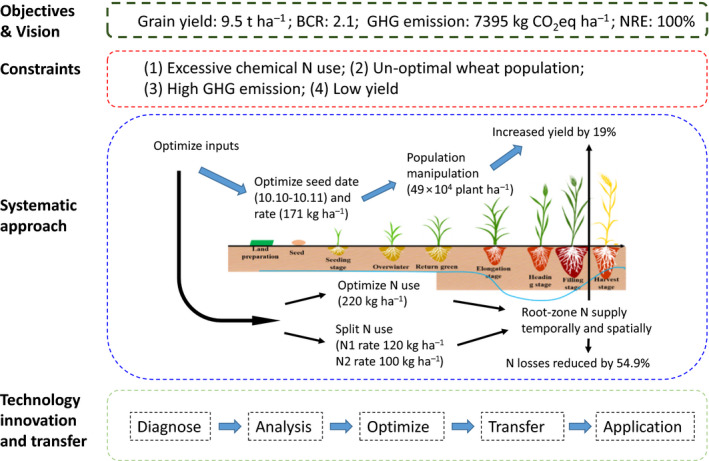
Concept model of integrated agronomy practices for multi‐objective optimization of wheat production employed by OPT smallholders. BCR: Benefit‐cost ratio, NRE: N recovery efficiency

Many studies have shown that participatory research, in which researchers and community members join together in a process of collaborative inquiry to address real‐world issues and practical problems, is an effective approach to handling the challenges of crop production (Bellon, 2001; Hoffmann et al., 2014; MacMillan and Benton, 2014). In this study, participatory research with Pareto‐based ranking was used to provide basic knowledge of optimal solutions to willing farmers in order to increase their problem‐solving skills. With clear Pareto optimal solutions to aim for, our research team and farmers jointly envisaged a desirable future of wheat production in which economic and environmental goals were optimized. The barriers and constraints to achieving this multi‐objective wheat production were jointly considered and analyzed. With the end vision in sight, scientists and farmers worked together to overcome the barriers they faced and jointly develop integrated agronomy practices for more optimal wheat production (Figure 6).

Our results indicate that, in particular, synergies existed between the grain yield and the N recovery efficiency and benefit‐cost ratio. Trade‐offs were found between GHG emissions and grain yield, N recovery efficiency and the benefit‐cost ratio. Synergy was also found between the N recovery efficiency and the benefit‐cost ratio. These factors are beyond the decision‐making capacity of smallholder farmers. A series of comparative field trials and demonstrations were conducted with the engagement of scientists and smallholder farmers. For instance, to persuade smallholders to reduce their chemical N fertilizer use, optimal N field trials were set up by smallholder farmers and scientists in farmers’ fields, and techniques for reducing N use were provided with guidelines. Through this approach, scientists transform from their traditional role of knowledge creators to becoming conduits between smallholder farmers to support collaboration at the interface of different communities (Chinseu et al., 2019; Snapp, Dedecker, & Davis, 2019).

In the present study, compared with farmers using typical practices (FP farmers), STB farmers reduced their sowing rate and chemical fertilizer use (Table 1; Figure 5). An integrated soil–crop management strategy, rather than solely technology alone, was employed. The optimal sowing rate and chemical N input level were the key points for achieving high yields and economic benefits with limited environmental impacts. On the North China Plain, due to the late maturity of maize, late sowing is common for wheat production. To maintain high tillering for high‐yield wheat, increasing the sowing rate and excessive chemical N use are common agronomic practices for smallholder farmers (Zhang et al., 2020). However, many studies have shown that excessive chemical N fertilizer inputs combined with a high sowing rate do not lead to high wheat production (Alzueta et al., 2012). This has caused massive N losses in cropland due to the limited N requirement in the early wheat growth stage without any corresponding benefits in terms of increased yield (Chen et al., 2018). In the present study, STB farmers reduced chemical N use and sowing rate to 246 kg/ha and 171 kg/ha, respectively (Figure 5). At the same time, wheat yield increased by 15.2% and GHG emissions decreased by 20.0%.

Furthermore, intensive, bottom‐up training rather than one‐time, top‐down training has been shown to be effective in improving the adoption of technology and improved agronomic practices by smallholder farmers (Zhao et al., 2016). The present study confirms this. The STB model in particular has the potential to disseminate innovative and participatory techniques and technology transfer for smallholder farmers (Zhang et al., 2016). In order to transform experimental science into smallholder farmer action, STBs conduct a series of demonstration field trials covering crop varieties, chemical fertilizer use, and pesticides based on farmer interests (Cui et al., 2018). Through this approach, the knowledge of integrated soil–crop management strategies is localized and more easily accepted by the smallholder farmers. The adoption rate has been shown to increase by 30% due to the novel approach of the STB program (Zhang et al., 2016). Our results here show that increased adoption rates have, in turn, led to more optimal farming outcomes for STB farmers versus other farmers using conventional practices (FP farmers).

### 4.3 Uncertainty analysis

There are some uncertainties surrounding the data used for our input parameters, which may to some extent impair the robustness and soundness of our results and conclusions. In the study, the coefficient of N flow, covering N harvested in grain (N_up_), NH_3_ volatilization (N_NH3_), N leaching (N_leach_), and N_2_O emissions (N_N2O_), was adopted from Chen et al. (2014). We also adopted their corresponding conversion factors to GHG emission from the same study. Generally speaking, N flow and GHG emissions are very difficult to obtain and are mainly determined in the fields plot conducted by scientists. However, it is possible to acquire a fair estimate by a comparison with other calculated N flow and GHG emissions in other regions. This was our approach, as noted in Section 2.2 and 2.4. Such uncertainties could be minimized by employing an intensive range of reference sources, including literature, questionnaires, and interviews. Local field experiment and monitoring may be an effective approach to acquire accurate data. In our cases, however, given the constraints of our study, we relied on the data provided by Chen et al. (2014).

## 5 CONCLUSIONS

In this study, we have shown that optimizing wheat production by smallholder farmers—both economically and environmentally—is possible by combining participatory research with Pareto optimality modeling. Under typical farmer practices (FP), the wheat yield and benefit‐cost ratio were 8.0 t/ha and 1.6, respectively, and GHG emissions were as high as 10,737 kg CO_2_eq ha^−1^ due to the low N recovery efficiency. Compared with these farmers, the grain yield and benefit‐cost ratio of Pareto optimal farmers (OPT) were 9.5 t/ha and 2.1, respectively; their N recovery efficiency and GHG emissions were 100% and 7,395 kg CO_2_eq ha^−1^, respectively. OPT farmers demonstrated higher grain yields and higher benefit‐cost ratios, along with lower GHG emissions and lower N inputs. Overall, STB farmers came closer to Pareto optimal levels than other (FP) farmers. Given the engagement of scientists and smallholder farmers through the STB model, grain yield and benefit‐cost ratio were improved by 15% and 22%, respectively, compared to FP farmers due to the improved N recovery efficiency. This resulted in a 20% reduction in GHG emissions for STB farmers. From the bottom‐up perspective, the corresponding adaptive agronomic practices, including optimizing chemical N use and the sowing rate, were employed by the STB farmers. The results indicate that multi‐objective optimization in wheat production can be achieved by modifying the appropriate agronomic practices through scientists and smallholder farmer engagement, with the help of Pareto optimality modeling to provide optimal, yet also realistic and attainable, end goals.

Connecting top‐down optimization and bottom‐up participatory implementation is a new frontier of sustainable agriculture research. The STB approach provides one way to combine this type of top‐down and bottom‐up strategizing. Through scientist–farmer experimentation, this approach offers a method for translating Pareto optimal solutions into sustainable crop production on the ground in smallholder field plots across China. Our study thus confirms that scientist–farmer engagement through STBs is an effective method for reducing the knowledge/action gap and optimizing wheat production by combining top‐down modeling with bottom‐up participatory approaches.

## CONFLICTS OF INTEREST

None declared.

## Supporting information

Table S1‐S2
