# Supplementary material for: Optimizing wheat production and reducing environmental impacts through scientist–farmer engagement: Lessons from the North China Plain
Source: Food Energy Secur. 2020 Nov 4;10(1):e255. doi: 10.1002/fes3.255 (PMC7988609; doi:10.1002/fes3.255)
Supplement: Supplementary file 1 — Table S1‐S2 [file FES3-10-e255-s001.docx]

| Table S1 Description and summary statistics for all variables under different farm types (FP, STB, and OPT) used in the study. Values, mean and range (minimum to maximum), were presented in the brackets. | | | | |
| --- | --- | --- | --- | --- |
|  |  |  |  |  |
| Variables | Description | FP | STB | OPT |
| Cultivated area (mu) | The area of wheat planted per household | 8（0.7-300） | 9.2（3-12） | 9.0（4-50） |
| Number of wheat variety | The number of genotypes used by smallholders | 3（1-5） | 3（1-4） | 2（1-3） |
| Sowing rate (kg ha^-1^) | The amount of seeds used in the per unit of lands | 202.5（22.5-600.0） | 172.5（150.0-262.5） | 185.25（142.5-262.5） |
| Chemical N use (kg N ha^-1^) | The amount of chemical N use per unit area | 260.4（157.5-516.2） | 246.0（198.8-288.0） | 221.3（159.0-270.8） |
| Fertilizer cost (CNY ha^-1^) | Fertilizer cost per unit of area | 3034.1（1350.0-8158.5） | 2633.2（2127.7-3389.7） | 2589.3（1860.0-3900.0） |
| Topdressing rate (kg N ha^-1^) | The amount of chemical N use for topdressing per unit area | 140.9（45.0-382.5） | 128.4（67.5-172.5） | 89（56.3-120.8） |
| Wheat yield (t ha^-1^) | Total wheat production per unit of area | 8.0（5.3-10.1） | 9.2（8.4-10.1） | 9.5（8.3-10.1） |
| Benefit-cost ratio | The ratio between costs and profit | 1.6（0.8-2.5） | 2（1.7-2.2） | 2.1（1.8-2.5） |
| N recovery efficiency (%) | The ratio between N uptake and N input | 75.9 (29.0-132.1) | 87.0 (68.5-109.4) | 100.0 (84.7-132.1) |
| GHG emission (kg CO_2_eq ha^-1^) | The amount of CO_2_eq emission per unit of area | 10737.3（4748.4-55311.8） | 8586.1（6243.2-11232.3） | 7395（4748.4-10007.9） |

Table S2 Uncertainties of N flows from wheat production in the three different groups (farmer practices (FP), Pareto optimization (OPT), and STB farmers (STB)) in Quzhou County in 2018.

| N flow (kg ha^-1^) | FP | | | STB | | | OPT | | |
| --- | --- | --- | --- | --- | --- | --- | --- | --- | --- |
|  | Median | (5^th^, 95^th^) | CVs | Median | (5^th^, 95^th^) | CVs | Median | (5^th^, 95^th^) | CVs |
| **N input** | 313 | (261.6, 473.9) | 0.04 | 266.9 | (311.1, 350.9) | 0.02 | 285.6 | (221.9, 333.6) | 0.1 |
| Chemical N | 250.1 | (198.8, 411.0) | 0.05 | 248.3 | (204, 288.0) | 0.03 | 222.8 | (159.0, 270.8) | 0.1 |
| N deposition | 28 | (14.9, 60.0) | 0.5 | 28 | (14.9, 60.0) | 0.5 | 28 | (14.9, 60.0) | 0.5 |
| Irrigation | 13 | (5.2, 15.0) | 0.3 | 13 | (5.2, 15.0) | 0.3 | 13 | (5.2, 15.0) | 0.3 |
| Seed | 2.8 | (2.4, 7.1) | 0.5 | 2.8 | (2.4, 7.1) | 0.5 | 2.8 | (2.4, 7.1) | 0.5 |
| Biological N | 15 | (11.4, 18.8) | 0.2 | 15 | (11.4, 18.8) | 0.2 | 15 | (11.4, 18.8) | 0.2 |
| **N output** | 260.5 | (225.1, 355.9) | 0.1 | 243.4 | (264.0, 280.3) | 0.02 | 267 | (239.8, 290.8) | 0.1 |
| N_2_O emission | 0.7 | (0.6, 2.3) | 0.2 | 0.5 | (0.7, 0.8) | 0.08 | 0.6 | (0.4, 0.7) | 0.2 |
| NH_3_ volatilization | 37.6 | (28.8, 64.9) | 0.1 | 31 | (37.4, 41.1) | 0.03 | 32.9 | (22.1, 41.1) | 0.2 |
| NH_3_ leaching | 21.6 | (16.2, 109.2) | 0.3 | 12.9 | (17.7, 25.5) | 0.1 | 14.8 | (8.4, 19.7) | 0.2 |
| N uptake | 200.5 | (179.5, 179.5) | 0.1 | 211.3 | (213.4, 191.8) | 0.04 | 219.8 | (194.2, 229.7) | 0.04 |
| N accumulation | 52.5 | (36.5, 118.0) | 0.4 | 18.7 | (42.6, 74.4) | 0.1 | 24.2 | (0, 42.8) | 0.7 |
